# Anti-hypercholesterolemic Effects and a Good Safety Profile of SCM-198 in Animals: From ApoE Knockout Mice to Rhesus Monkeys

**DOI:** 10.3389/fphar.2018.01468

**Published:** 2018-12-13

**Authors:** Rinkiko Suguro, Siyao Chen, Di Yang, Zunyuan Yang, Lei Miao, Weijun Wu, Wen Zeng, Xinhua Liu, Yi Zhun Zhu

**Affiliations:** ^1^Shanghai Key Laboratory of Bioactive Small Molecules, Department of Pharmacology, School of Pharmacy, Fudan University, Shanghai, China; ^2^School of Pharmacy, Macau University of Science and Technology – State Key Laboratory of Quality Research in Chinese Medicine, Taipa, Macau; ^3^Department of Cardiac Surgery, Guangdong General Hospital, Guangdong Cardiovascular Institute, Guangdong Academy of Medical Sciences, Guangzhou, China; ^4^Sichuan Primed Co., Ltd., Chengdu, China

**Keywords:** leonurine, SCM-198, atherosclerosis, dyslipidemia, Rhesus monkeys

## Abstract

Although several lipid-lowering agents have been introduced for the treatment of atherosclerosis (AS), currently marketed medications have not solved the problem completely. This study aims to investigate the effects of leonurine (SCM-198) on dyslipidemia in mammals with ApoE knockout (ApoE^-/-^) mice, New Zealand white rabbits and senile Rhesus monkeys fed with high fat diet were dosed daily with leonurine or atorvastatin. The serum total cholesterol (TC), triglyceride (TG), low density lipoprotein (LDL), and high-density lipoprotein (HDL) were determined. Moreover, in Rhesus monkeys, bodyweight, arterial ultrasound of right common carotid artery, Apolipoprotein A1 (ApoA1) and ApoB levels, hematologic and toxicological examinations were detected. Serum TC and TG in both mice and rabbits were significantly reduced by SCM-198 and atorvastatin. In the 10 mg/kg SCM-198 group of monkeys, maximum TC reduction of 24.05% was achieved at day 150, while 13.16% LDL reduction achieved at day 60, without arterial morphologic changes or adverse events. Atorvastatin (1.2 mg/kg) showed similar effects as SCM-198 in improving lipid profiles in monkeys, yet its long-term use could induce tolerance. Furthermore, leonurine suppressed genes expression of fatty acid synthesis, such as fatty acid synthase (FASN), stearoyl-CoA desaturase (SCD-1), sterol regulatory element-binding protein (SREBF) in liver in high fat diet feeding ApoE^-/-^ mice. SCM-198, with a reliable safety profile, is of high value in improving lipid profiles in mammals, providing an alternative to a substantial population who are statin-intolerant.

## Introduction

Atherosclerosis (AS) remains a global health burden to date (Mizia-Stec et al., 2003; Stark, 2015), in the developed countries a high mortality rate of approximately 50% was reported among patients with AS or with AS-associated complications such as myocardial infarction and cerebrovascular events (Lusis, 2000; Mallika et al., 2007). Meanwhile, the incidence of AS is rising in acceleration in the developing world, particularly in China, the most populated country on earth.

Atherosclerotic lesions compromising coronary artery, cerebral artery or internal carotid artery could lead to acute ischemic event, vascular thrombosis and tissue infarction among many others, which prone to be life-threatening. Moreover, AS is a multi-factorial disease attributable to the interaction between genetic factors and environmental stimulants, which include dyslipidemia, hypertension, diabetes, smoking, obesity, and so on. In particular, low density lipoprotein (LDL) has been established as a major contributing factor to AS (Steinberg, 2005, 2006). Also, oxidative stress and inflammatory responses as well as their interactions play key roles in the development of AS (Hulsmans and Holvoet, 2010). Endothelial cells (ECs) malfunction has been reported as the initiating step of AS development (Simionescu, 2007).

Several irritants could induce ECs dysfunction resulting in increased perviousness of vascular endothelium, enabling LDL accumulation beneath endothelium, promoting expression of endothelial adhesive molecules, such as selectin and intercellular adhesive molecules, thus further promoting endothelial adhesion of monocytes, T lymphocytes, and platelets. Monocyte migration and differentiation as macrophage in endothelium would further initiate the oxidase system in vasculatures and LDL molecules being oxidized into pro-inflammatory cytokine, ox-LDL. Ox-LDL could promote in ECs and vascular smooth muscle (VSMC) the synthesis of inflammatory regulators and chemokines including MCP-1 and IL-8, advocating leukocyte adhesion and inflammation; furthermore, ox-LDL could stimulate inflammatory cells and promote in vascular cells the secretion of inflammatory cytokines and growth factors such as platelet derived growth factors, fibroblast growth factors and transforming growth factors, contributing to VSMCs migration and proliferation (Hoff and O’neil, 1991). Scavenger receptor mediated phagocytosis of ox-LDL could induce cholesterol accumulation and foamy-like transformation in macrophages and VSMCs with increased generation of collagen (Libby, 2002). Malignant cycle of inflammation and oxidative stress would certainly accelerate the formation of fibro-fatty plaques in AS.

Many lipid-lowering agents have been introduced for the prevention and treatment of AS. However, currently marketed medications including atorvastatin have not solved the problem completely. Notably, a substantial population cannot benefit from statin therapy due to intolerance. Therefore, introduction of novel effective anti-atherosclerotic drug with reliable safety profile as an alternative is in need.

*Herba leonuri*, also known as motherwort herb, is a kind of commonly used traditional Chinese medicine (TCM) with the effect of reducing blood stasis, regulating menstrual disorder, alleviating edema, etc. Leonurine (SCM-198), containing alkaloid constituents of *Herba leonuri*, has been reported as a competitive drug candidate for the treatment of myocardial ischemia and cardiac insufficiency in rats (Liu et al., 2009; Loh et al., 2010; Shi et al., 2011). It is suggested by several studies conducted in rat models that leonurine demonstrates potent protective effects in both the cardiovascular system and cerebrovascular system, with strong potential for the prevention and treatment of AS (Liu et al., 2010, 2013). Therefore, the present study aims to investigate the novel anti-atherosclerotic effect of SCM-198 using atherosclerotic models of ApoE knockout (ApoE^-/-^) mice, New Zealand white rabbits and senile Rhesus monkeys fed with high cholesterol diet, respectively.

## Materials and Methods

### Chemicals and Reagents

SCM-198 (manufacture no: MC0948-055-1-2) was synthesized and then purified (over 98%) by silica gel chromatography. Atorvastatin (manufacture no: A150101) was purchased (Haizheng Pharmaceutical Co., Ltd., Zhejiang, China) and freshly dissolved in distilled water before administration. The levels of LDL, high-density lipoprotein (HDL), triglyceride (TG), and total cholesterol (TC) were determined by automatic biochemical analyzer. Antibodies stearoyl-CoA desaturase (SCD-1) and fatty acid synthase (FASN) were purchased from Cell Signaling Biotechnology (Danvers, MA, United States). Other reagents were purchased from Sigma-Aldrich (St. Louis, MO, United States) unless otherwise stated.

### Animals

Male C57BL/6J (SPF, 8–10 weeks) mice and ApoE knockout (ApoE^-/-^, SPF, 8–10 weeks) mice were purchased from Beijing HuaFuKang Biotechnology Co., Ltd. (certification number: SCXK, Peking, 2014-0008). ApoE^-/-^ mice fed by Western diet (21% fat and 19.8% protein, D12079B, Research Diet Inc., New Brunswick, NJ, United States) were randomly divided into vehicle (ApoE^-/-^, distilled water, *i.g.*), low dose leonurine (10 mg/kg/day, *i.g.*), high dose leonurine (20 mg/kg/day, *i.g.*), and atorvastatin (positive control, 3 mg/kg/day, *i.g.*) (*n* = 10 for each group). Mice were sacrificed after 4 weeks and 0.5 ml blood was collected for hematological examinations.

Male New Zealand White Rabbits (1.8–2.2 kg) were purchased from Shanghai Jiagan Biotechnology Co., Ltd. (certification number: SYXK, Shanghai, 2013-0087). Rabbits were randomly divided into control (normal diet, distilled water, *i.g.*), vehicle (high fat diet, distilled water, *i.g.*), low dose leonurine (high fat diet, 4 mg/kg/day, *i.g.*), middle dose leonurine (high fat diet, 8 mg/kg/day, *i.g.*), high dose leonurine (high fat diet, 16mg/kg/day, *i.g.*), and atorvastatin (positive control, high cholesterol diet, 2.5 mg/kg/day, *i.g.*) (*n* = 6 for each group). Rabbits were starved at week 12 before blood collection for hematological examinations.

Eight male Western Sichuan subspecies *Rhesus* monkeys with Animal Quarantine Conformity Certificate were purchased from Yaan Biological Technology Co., Ltd. (certification number: SCXK, Sichuan, 2014-027). Monkeys with serum TC ≥ 4.1 mmol/L, LDL-C ≥ 1.1mmol/L, and more than one intima-media thickness (IMT) ≥ 0.5 mm were enrolled in the present experiment and divided into three groups; vehicle (*n* = 2), SCM-198 (*n* = 3, 10.0 mg/kg), atorvastatin (*n* = 3, 1.2 mg/kg). Measurement of bodyweight was conducted twice prior to treatment, at day 8, day 1 (defined as baseline), every 30 days, and after washout, in total nine times. Experiment process included 28 days of quarantine period, 178 days of drug administration, washout period for 14 days with the first day of drug administration defined as day 0 (Supplementary Figure S1).

All animals were free to receive food and water and managed on standard conditions according to the “Guide for the Care and Use of Laboratory Animals” published by the US National Institute of Health (NIH). All animals and the experimental protocol conformed to the Animal Welfare Act Guide for Use and Care of Laboratory Animals, and were approved by Institutional Animal Care and Use Committee (IACUC), School of Pharmacy, Fudan University, China, and the IRB approval number is 2015-09-ZYZ-01.

### Serum Lipid Profiles

Animal blood was collected and centrifuged at 3000 rpm/min for 10 min, and then supernatant was harvested. Determination of serum TC, TG, LDL, and HDL, ApoA1, ApoB and blood glucose was conducted following manufacture’s guidelines using enzyme analyzer and auto biochemical analyzer. Determination of lipid profiles was conducted for three times prior to drug administration (respectively at day 21, day 12 and day 1, with day 1 as baseline) and post drug administration at day 15, day 30, day 60, day 90, day 120, day 150, day 178, and day 194, in total 11 times.

### Artery Morphology Detected by Ultrasound

Artery morphology was examined by sonosite M-turbo color Doppler ultrasound both prior to drug treatment and post treatment. The changes of right common carotid artery (RCCA), right common carotid artery bifurcation (RBIF), left common carotid artery (LCCA), left common carotid artery bifurcation (LBIF), and abdominal aorta (AO) were analyzed.

### Lab Examinations

Animals were starved before blood collection. Serum was harvested by centrifugation at 8,000 rpm for 5 min. Biochemistry parameters were detected by Beckman CX4 auto biochemical analyzer and hematological changes determined by McDonnell Douglas Nick CA620 blood cell analyzer.

### Quantitative Real Time RT-PCR Assay and Western Blotting

Total RNA was extracted from the liver tissues with TRIzol reagent following the manufacturer’s instructions. Total RNA of each sample was reverse-transcribed into first-strand cDNA and amplified with a PrimeScript^TM^ 1st Strand cDNA Synthesis Kit (Takara) according to the manufacturer’s directions. PCR parameters were set as follows: denaturation (95°C for 15 s); annealing (56°C for 45 s); extension (55°C for 15 s) and number of cycles (40). The primers used in this experiment were shown in Supplementary Table S1.

Liver tissue proteins were lysed in RIPA buffer (Pierce, Rockford, IL, United States) containing protease inhibitor cocktail (Sigma, St. Louis, MO, United States). Whole lysates samples were separated by sodium dodecyl sulfate-polyacrylamide gel electrophoresis, and then were transferred onto nitrocellulose membranes for immunoblot analysis. The membranes were incubated with the indicated primary antibodies, and then incubated with an IRDye 800 anti-rabbit secondary antibody for 1 h at room temperature. Finally, the membrane was imaged in an Odyssey CLx Imaging System (LI-COR, Lincoln, NE, United States).

### Statistical Analysis

All data are presented as means ± SEM and statistical analysis was conducted using the analysis of variance (ANOVA or Student’s test). *Post hoc* pairwise comparisons were performed using Prism graph. *P*-value < 0.05 was considered statistically significant.

## Results

### Leonurine Reduced Lipid Profiles in ApoE^-/-^ Mice

**FIGURE 1:**
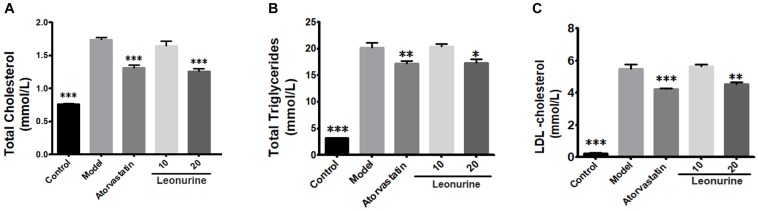
Changes of lipid profiles of ApoE^-/-^ mice treated by different dose of SCM-198 and atorvastatin, respectively. The changes of total cholesterol **(A)**, total triglycerides **(B)**, LDL-cholesterol **(C)** in high fat diet mice were examined by automatic biochemistry analyzer. Model: high fat diet; all data are presented as means ± SEM and statistical analysis was conducted using the analysis of variance (ANOVA or Student’s test), *n* = 10; ^∗^*p* < 0.05, ^∗∗^*p* < 0.01, ^∗∗∗^*p* < 0.001 vs. model group.

The changes of lipid profiles in atherosclerotic ApoE^-/-^ mice treated, respectively, by different doses of SCM-198 were determined by automatic biochemistry analyzer. Compared with the normal diet group of mice, serum levels of TC, TG and LDL in the high fat diet group of mice was markedly increased (*p* < 0.001). Meanwhile, serum levels of TC, TG, and LDL in both atorvastatin group and 20 mg/kg leonurine group were reduced as compared with the high fat diet group. Nevertheless, leonurine failed to promote HDL levels in high fat diet mice (data not shown) (Figure 1).

### Leonurine Reduced Lipid Profiles in Hypercholesterolemia Rabbits

**FIGURE 2:**
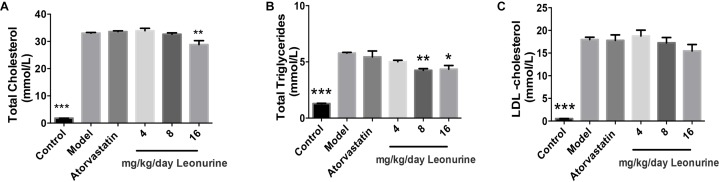
Changes of lipid profiles of hypercholesterolemia New Zealand white rabbits treated by different dose of SCM-198 and atorvastatin, respectively. The changes of total cholesterol **(A)**, total triglycerides **(B)**, LDL-cholesterol **(C)** in hypercholesterolemia rabbits were determined by automatic biochemistry analyzer. Model: high fat diet; all data are presented as means ± SEM and statistical analysis was conducted using the analysis of variance (ANOVA or Student’s test), *n* = 6; ^∗^*p* < 0.05, ^∗∗^*p* < 0.01, ^∗∗∗^*p* < 0.001 vs. model group.

The changes of lipid profiles in high fat New Zealand white rabbits treated, respectively, by different doses of leonurine and atorvastatin were determined by automatic biochemistry analyzer. Compared with the normal diet group of rabbits, serum levels of TC and TG in the high fat diet group of rabbits were markedly increased (*p* < 0.001), whereas 16 mg/kg leonurine reduced serum levels of TC (*p* < 0.01) and TG (*p* < 0.05) in high fat diet rabbits. Also, 8 mg/kg SCM-198 reduced TG levels in the high fat rabbits (Figure 2). Compared with normal diet rabbits, serum levels of LDL in rabbits of the high fat diet group were increased significantly (*p* < 0.001), while leonurine failed to reduce LDL.

By far, our data showed potent anti-atherosclerotic effects of leonurine in hypercholesterolemia mice and rabbits.

### Effect of SCM-198 on the Bodyweight, Lipids, and Lipoproteins of Rhesus Monkeys

**FIGURE 3:**
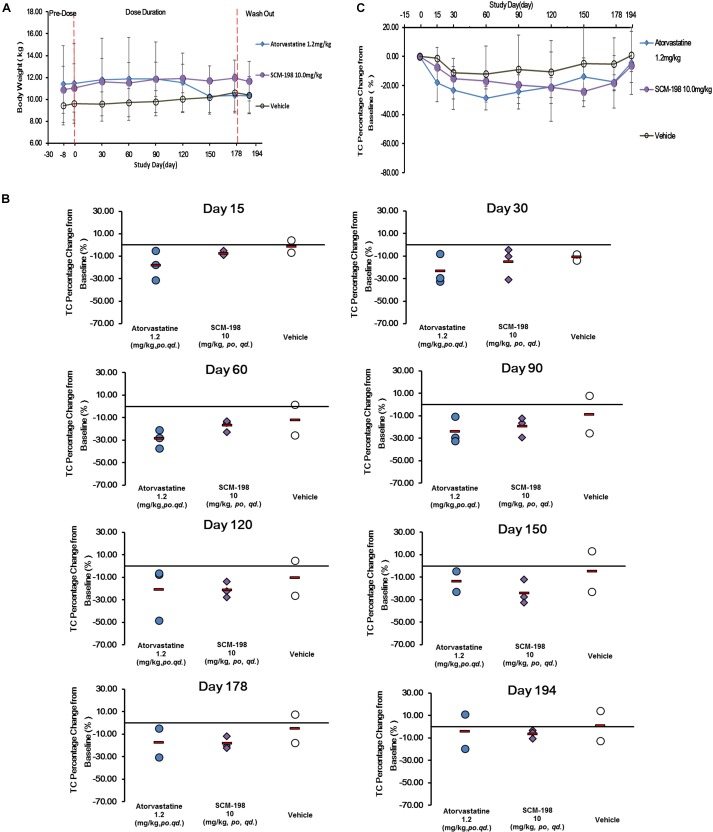
Changes of bodyweight and total cholesterol (TC) levels in hypercholesterolemia Rhesus monkeys, respectively, treated by SCM-198 and atorvastatin continuously for 178 days. **(A)** Comparison of the changes of bodyweight in different groups of hypercholesterolemia Rhesus monkeys, respectively, treated by SCM-198 and atorvastatin continuously for 178 days; **(B)** The percentage changes of serum TC levels at different time points in hypercholesterolemia Rhesus monkeys; **(C)** The percentage changes of serum TC levels in hypercholesterolemia Rhesus monkeys (*n* = 3 for SCM-198 and atorvastatin group, *n* = 2 for vehicle group).

The impact of different drugs on the bodyweight of monkeys was shown in Figure 3A. Compared with baseline on day 1, the bodyweight of monkeys between different groups showed no statistically significant difference.

The effect of SCM-198 on serum TC levels of in hypercholesterolemia monkeys were shown in Figure 3B. Serum TC levels in the control monkeys were comparatively stable without fluctuations, suggesting the stability of the animal model. The analysis of the effect of 1.2 mg/kg atorvastatin on serum TC level showed that after 15 days of treatment, TC level was markedly reduced, which continued till day 60 when a platform phase was observed; TC level was markedly increased after washout. Compared with the baseline value, the percentage reduction of TC achieved 18.02% at day 15, with maximum reduction of 28.64% at day 60. The percentage reduction of TC was 17.51% at day 178 and 4.39% after washout. Our data suggested that at the dose of 1.2 mg/kg, atorvastatin showed potent TC reducing effect. 10 mg/kg SCM-198 group showed a significant decrease of TC level by day 15, which continued till the end of drug treatment. Compared with the baseline value, a 7.44% of TC reduction was observed at day 15, with maximum reduction of 24.05% observed at day 150, which, however, decreased to 6.42% after washout, suggesting that at the dose of 10 mg/kg SCM-198 showed potent TC reducing effect, similar as 1.2 mg/kg atorvastatin (Figure 3C).

**FIGURE 4:**
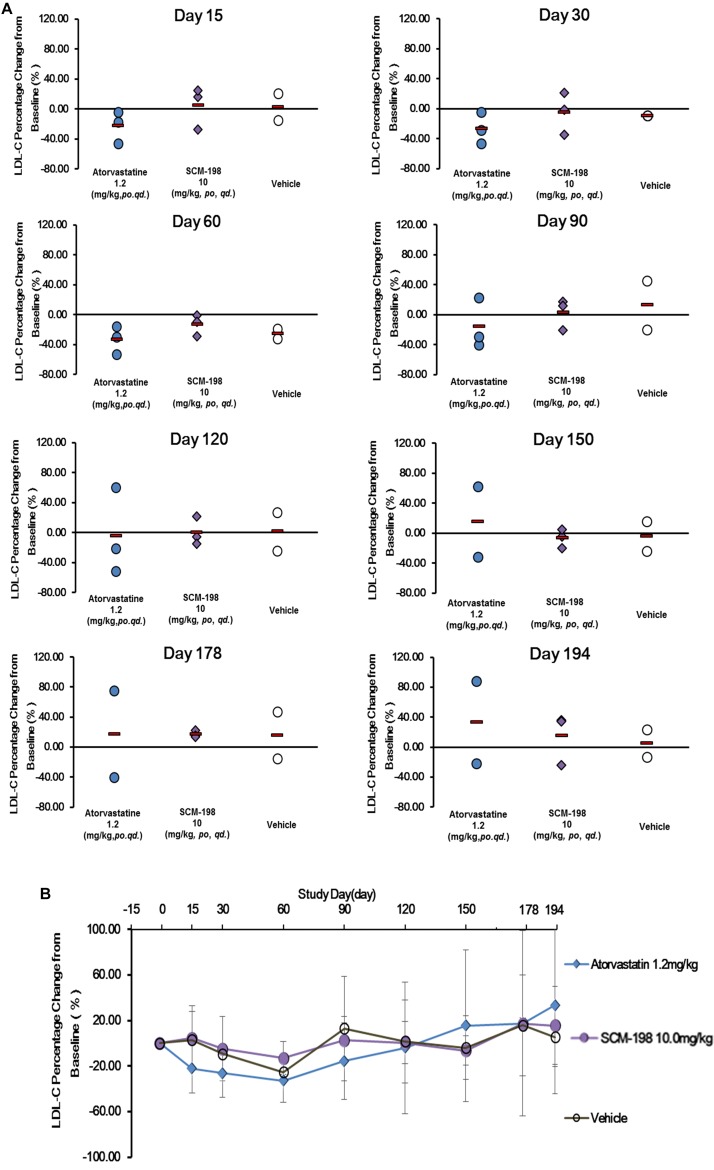
Comparison of the percentage change of low density lipoprotein cholesterol (LDL-c) levels in hypercholesterolemia Rhesus monkeys, respectively, treated by SCM-198 and atorvastatin continuously for 178 days. **(A,B)** The percentage change of LDL-c in hypercholesterolemia Rhesus monkeys, respectively, treated by SCM-198 and atorvastatin examined at different time points (*n* = 3 for SCM-198 and atorvastatin group, *n* = 2 for vehicle group).

The effect of SCM-198 on the LDL-c levels in hypercholesterolemia Rhesus monkeys were shown in Figure 4. The serum LDL-c levels in the control group showed slight fluctuations during the experiment, which was of no statistical significance. The analysis of the effect of 1.2 mg/kg atorvastatin on the serum LDL-c level demonstrated that after 15 days of treatment, LDL-c level was markedly reduced, which continued till day 60 when a maximum reduction was observed; reduction of LDL-c level was maintained till the end of the experiment with a slight rebound after washout. After 30 days treatment of 10 mg/kg SCM-198, we first found reduction of LDL-c level, which continued till day 60 when a maximum reduction found (Figure 4A). Compared with the baseline, the percentage reduction of LDL-c achieved 4.89% at day 30, with a maximum reduction of 13.16% at day 60 and slight rebound after washout (Figure 4B).

**FIGURE 5:**
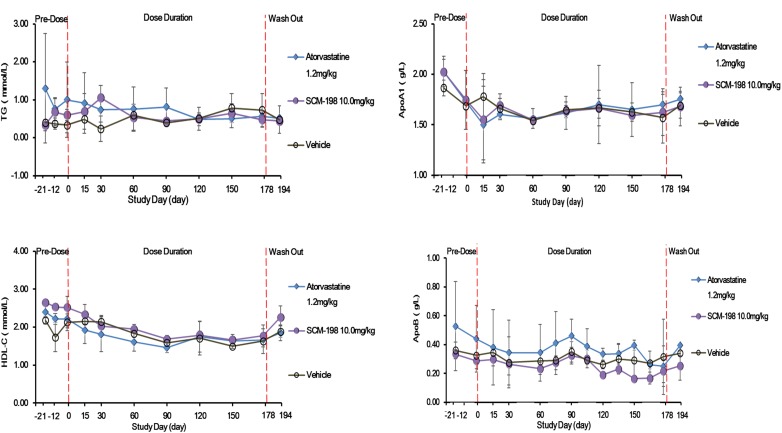
The changes of levels of triglycerides (TG), high density lipoprotein cholesterol (HDL-c), apolipoprotein A1 (ApoA1), and ApoB in hypercholesterolemia Rhesus monkeys, respectively, treated by SCM-198 and atorvastatin continuously for 178 days. Comparison of the levels of TG; HDL-c; ApoA1; and ApoB between different groups of hypercholesterolemia Rhesus monkeys, respectively, treated by SCM-198 and atorvastatin continuously for 178 days (*n* = 3 for SCM-198 and atorvastatin group, *n* = 2 for vehicle group).

The serum TG, HDL-c, ApoA1, and ApoB level of the control group showed no significant change during the experiment and also presented no statistically significant difference as compared with the SCM-198 group (Figure 5).

### SCM-198 Showed No Effect on Artery Morphology

The effect of SCM-198 on the artery morphology of high fat Rhesus monkeys treated by SCM-198 for 178 days was determined. In the control group, no significant changes of RCCA, RBIF, LCCA, LBIF, or AO were observed during the experiment. Compared with prior to treatment, no statistically significant changes of RCCA, RBIF, LCCA, LBIF, or AO were found post treatment for 178 days in the 1.2 mg/kg atorvastatin group. Also, results in the 10 mg/kg SCM-198 group were similar as atorvastatin that in comparison to prior to treatment, no changes of RCCA, RBIF, LCCA, LBIF, or AO were found post treatment (Supplementary Figure S2 and Supplementary Table S2).

### Hepatic Toxicity and General Safety

**FIGURE 6:**
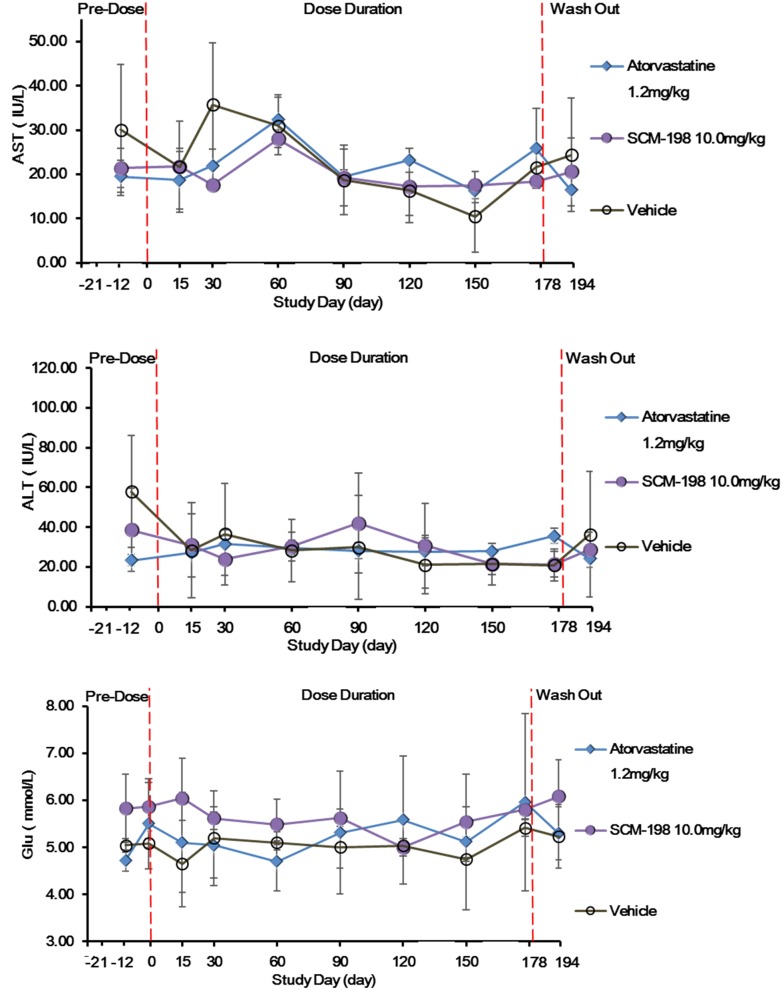
The effect of SCM-198 treatment on blood glucose (GLU) level and hepatic enzymes of hypercholesterolemia Rhesus monkeys. Comparison of the levels of AST, ALT, and GLU between different groups of hypercholesterolemia Rhesus monkeys, respectively, treated by SCM-198 and atorvastatin continuously for 178 days (*n* = 3 for SCM-198 and atorvastatin group, *n* = 2 for vehicle group).

The effect of sustained SCM-198 treatment for 178 days on the hepatic enzymes of hypercholesterolemia Rhesus monkeys was determined. No significant changes of the level of ALT, AST, or glucose (GLU) were observed in the SCM-198 group during the experiment. The impact of SCM-198 treatment for 178 days on the glucose level of high fat Rhesus monkeys was analyzed and no change of statistical significance between different groups during the experiment was observed (Figure 6). Biochemical examinations revealed that all biochemical index prior to treatment was of no significant difference as compared with post treatment for 178 days; 10 mg/kg SCM-198 had no adverse effects, with biochemical index maintained within physiologic levels. Consistent with biochemical findings, hematologic examinations showed that 10 mg/kg SCM-198 had no effect on hematologic parameters; no change of hematological index was found between different groups prior to treatment and post treatment (Supplementary Tables S3, S4).

### Leonurine Suppressed the Synthesis of Fatty Acids in Liver

**FIGURE 7:**
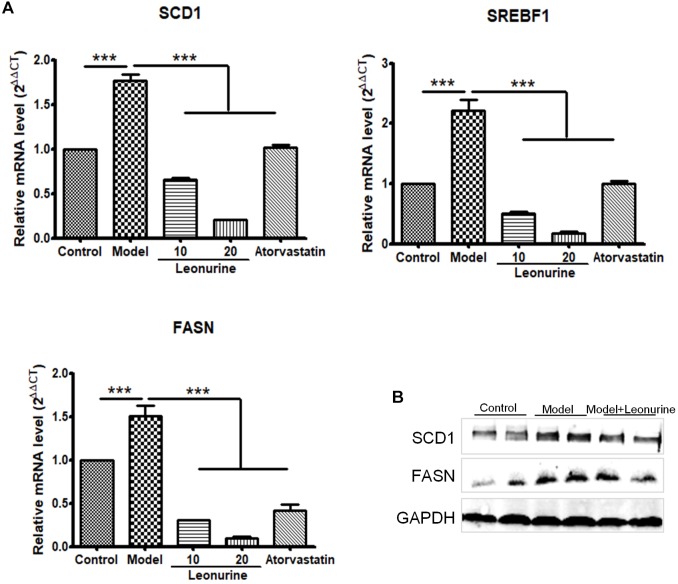
Leonurine suppressed genes expression of fatty acid synthesis in liver in high fat diet feeding ApoE^-/-^ mice. **(A)** qRT-PCR analysis of *FASN*, *SCD-1* and *SREBF1* from mRNA isolated from livers of C57 and ApoE^-/-^ mice fed a control or high fat diet for 4 weeks. Values are given as means ± SEM for *n* = 10; ^∗∗∗^*p* < 0.001 vs. Model group. **(B)** FASN and SCD-1 protein expression were detected by western blotting. Control, control diet; Model, high fat diet; FASN, fatty acid synthase; SCD-1, stearoyl-CoA desaturase 1; SREBF, sterol regulatory element-binding protein.

The molecular mechanism involving leonurine protecting against AS was studied. Based on our current results, leonurine can dramatically decrease the mRNA expression of FASN, SCD-1, sterol regulatory element-binding protein (SREBF) in liver in high fat diet feeding ApoE^-/-^ mice (Figure 7A), consistent to this, FASN and SCD-1 protein level showed a decrease in mice treated with leonurine (Figure 7B), thereby reducing the synthesis of fatty acid in liver, so help to reduce blood lipid levels.

## Discussion

Atherosclerosis and its associated vascular complications remain a severe health burden global wide, with costly medical expenses. Early diagnosis and intervention is essential for the prevention of AS and associated cardiovascular and cerebrovascular events. According to epidemic data, AS patients are three times more likely to have cardiovascular diseases, myocardial infarction and cerebrovascular accidents. Given the important role of dyslipidemia in the progression of AS, risk reduction strategy mainly highlights the improvement of lipid profile. Reduced LDL-c level has been proved to be highly effective in lowering the incidence of major cardiovascular events as reported in several large trials (Baigent et al., 2005; Cholesterol Treatment Trialists et al., 2010). For this reason, reducing LDL-c, increasing HDL-c, and reducing TGs have been established as fundamental means of reducing cardiovascular morbidity and mortality (Jacobson et al., 2015; Perez de Isla et al., 2017).

Statin, inhibitor of 3-hydroxy-3-methylglutaryl coenzyme A reductase, is a fundamental treatment against dyslipidemia and has been incorporated into practice guidelines as primary and secondary prevention of AS. Unfortunately, a large population cannot benefit from statin therapy due to severe adverse events, heart failure, or hemodialysis (Wanner et al., 2005; Joy and Hegele, 2009; Fishbane et al., 2013). The introduction of novel anti-atherosclerotic agents with favorable tolerance and safety profile would offer an alternative for patients who cannot tolerate or reach LDL-c therapy goals using only statins.

Leonurine (SCM-198) is an active component extracted from traditional Chinese herbs, *Herba leonuri*, also named as Motherwort herb, which has been proved by few studies to be an agent with protective effects in multiple systems particularly in the cardiovascular system and hemodynamic system (Liu et al., 2010, 2013). Another study revealed that, SCM-198 could increase lymphatic contraction frequency and dilate lymphatic vessels in rat mesentery, thereby improving lymphatic micro-environment (Chen and Kwan, 2001). Given the molecular structure of SCM-198, it can be deduced that its modified base might be associated with its potent activity and effect of anti-platelet aggregation. We speculate that modification of this base into amino base might further augment its effects. Moreover, SCM-198 has demonstrated marked anti-inflammatory effects (Liu et al., 2018). It was reported that SCM-198 could improve local circulation, attenuate inflammatory effusion, and accelerate inflammatory absorption in dysmenorrhea, thereby significantly relieving pain. Of note that the protective functions of SCM-198 on the cardiovascular system were mainly associated with its effects of improving myocardial function, increasing coronary circulation, and relieving myocardial ischemia (Zhang et al., 2012; Liu et al., 2013; Feng et al., 2016).

Based on previous studies of multiple cardiovascular protective effects of SCM-198, ApoE^-/-^ mice with high cholesterol diet was used to build AS model and results showed that SCM-198 could markedly reduce serum total TC, TG, LDL, while no significant effect on HDL observed; meanwhile, similar lipid-reducing effect of atorvastatin was observed. Furthermore, we tested our thesis using New Zealand white rabbits fed with high fat diet, different doses of SCM-198 could reduce TC and TG, although the levels of LDL and HDL appear not regulated by SCM-198. The slight discrepancy of results between the two animal experiments conducted inspires the speculation that both mice and rabbits are not genetically proximate to human beings and thereby probably share quite different metabolic system from humans.

Primates, with similar genetics, cardiovascular function and lipid metabolism as human, could better mimic the pathophysiologic process of AS in humans. For this reason, further experiments introduced senile Rhesus monkeys fed with high fat diet for 178 days in order to build the model of AS, and the efficacy and safety profile of SCM-198 and atorvastatin were analyzed. Statins could reduce cholesterol synthesis via inhibiting HMG-CoA reductase and therefore are commonly used in hyperlipidemia patients for reducing levels of TC and LDL; of note that its efficacy and dose are positively associated. The present study presented that 1.2 mg/kg atorvastatin could effectively reduce serum levels of TC and LDL in senile Rhesus monkeys, with maximum reduction at 60 days; however, longer administration of this single regimen of atorvastatin suggested intolerance to some degree. No adverse events of hepatic damage as suggested by changes of AST and ALT levels were found in monkeys treated with same dose of atorvastatin. SCM-198 could improve hemodynamics through promoting blood flow and erythrocyte deformity, attenuating the condensed aggregated pathologic microcirculation caused by high cholesterol level. The present study demonstrated that 10 mg/kg SCM-198 could reduce LDL levels with maximum reduction at 60 days, yet of note that longer treatment showed rebound in LDL levels. Maximum reduction of TC was observed at 150 days of SCM-198 treatment. No adverse events of hepatic damage in monkeys treated by 10 mg/kg SCM-198 was observed. Moreover, treated with 10 mg/kg SCM-198 for 178 days, serum TC levels in hypercholesterolemic monkeys were markedly reduced. Atorvastatin shared similar potency and efficiency as SCM-198, which had weaker effect on LDL reduction as compared with atorvastatin.

Few studies revealed that statins could not only improve lipid profiles but also stabilize atherosclerotic plaques by improving endothelial function, inhibiting inflammatory responses, reducing lipid deposition on endothelium, inhibiting formation of foam cells. Interestingly, in the present study, hypercholesterolemic monkeys in the atorvastatin group showed no change of the artery IMT after 6 months of treatment. SCM-198, with multiple effects including invigorating blood and removing blood stasis, also showed no effect on artery IMT in hypercholesterolemic monkeys after 6 months of treatment. Worth notably, increased thickness of artery IMT determined by ultrasound has been shown to be directly associated with an increased risk of myocardial infarction and stroke in older adults without a previous history of cardiovascular disease (Baldassarre et al., 2000; Juonala et al., 2004; Zeng et al., 2015). Further study of the impact of SCM-198 on atherosclerotic plaque is in need.

The liver, as the primary site for the synthesis of fatty acids and cholesterol, plays an important role in maintaining lipid homeostasis. Fatty acids are transferred to TGs in liver cells and secreted into the circulation system for peripheral tissue utilization or storage through very low density lipoprotein (VLDL) (Fabbrini et al., 2010). The liver lipid synthesis process is mainly controlled by sterol regulatory element-binding protein (SREBP1, expressed by SREBF1 gene) (Debose-Boyd and Ye, 2018). SREBP1 promotes the synthesis of fatty acids through activation of downstream target genes, such as acetyl-CoA carboxylase 1 (ACC1), FASN, SCD-1 and glycerin-3-phosphate acyltransferase (GPAT) (Wang et al., 2009; Strable and Ntambi, 2010). Inhibiting the expression of SREBF1 and downstream target genes can reduce the synthesis of liver fatty acids, which can help reduce lipid levels. Our current results showed leonurine could dramatically decrease the expression of SREBP1, FASN, and SCD1 in hypercholesterolemic mice, suppressing the synthesis of fatty acids in liver.

The strength of the present study is that we successfully replicated atherosclerotic models of mice, rabbits and monkeys and proved in these models the anti-atherosclerotic effects of SCM-198, which would provide novel evidence rooting for the potent cardiac protective effect of SCM-198 as well as offer experimental basis for future clinical application. Some limitations of this study warrant mention that our samples of Rhesus monkeys were small, which might reduce the statistical power of our data. The present findings still strongly suggest that this potent SCM-198 is of high value in reducing lipids in atherosclerotic animals with dyslipidemia.”

**FIGURE 8:**
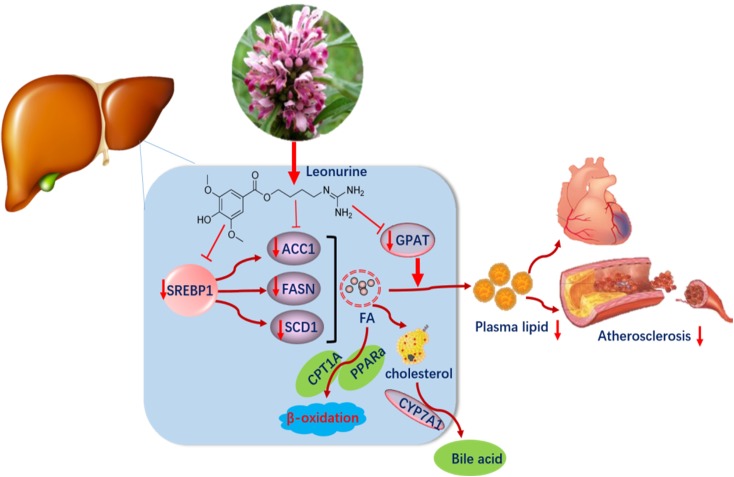
Leonurine downregulated the expression of SREBP1, FASN, and SCD1 in the liver, so suppressed the synthesis of fatty acid and lowered plasma lipid levels.

We conclude that SCM-198 exerts potent lipid-lowering effects and the molecular mechanisms might involve that SCM-198 could dramatically decrease the synthesis of fatty acids in liver, and thus reducing blood lipid levels. Identifying novel agent such as SCM-198 in treating dyslipidemia is of significant value in implementing preventive measures against AS and coronary artery diseases (Figure 8).

## Author Contributions

Data collection and analysis were provided by RS and SC. Scientific editorial assistance of the article was provided by LM. Clinical insights and review were provided by WW and DY. The study was designed and supervised by XL and YZ. All authors commented and approved the submitted manuscript.

## Conflict of Interest Statement

WZ was employed by Sichuan Primed Co., Ltd. The remaining authors declare that the research was conducted in the absence of any commercial or financial relationships that could be construed as a potential conflict of interest.
