# Supplementary material for: Anti-hypercholesterolemic Effects and a Good Safety Profile of SCM-198 in Animals: From ApoE Knockout Mice to Rhesus Monkeys
Source: Front Pharmacol. 2018 Dec 13;9:1468. doi: 10.3389/fphar.2018.01468 (PMC6300478; doi:10.3389/fphar.2018.01468)
Supplement: Supplementary file 1 [file Data_Sheet_1.docx]

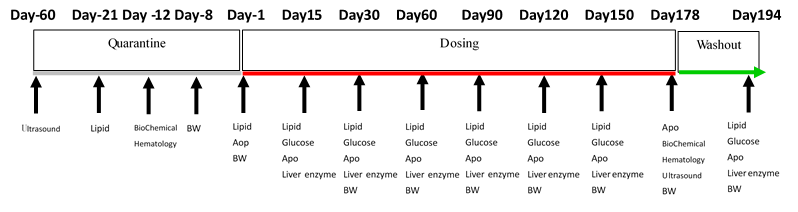


**Figure S1. Work chart of drug administration.** Experiment process included 28 days of quarantine period, 178 days of drug administration, washout period for 14 days with the first day of drug administration defined as day 0.

**
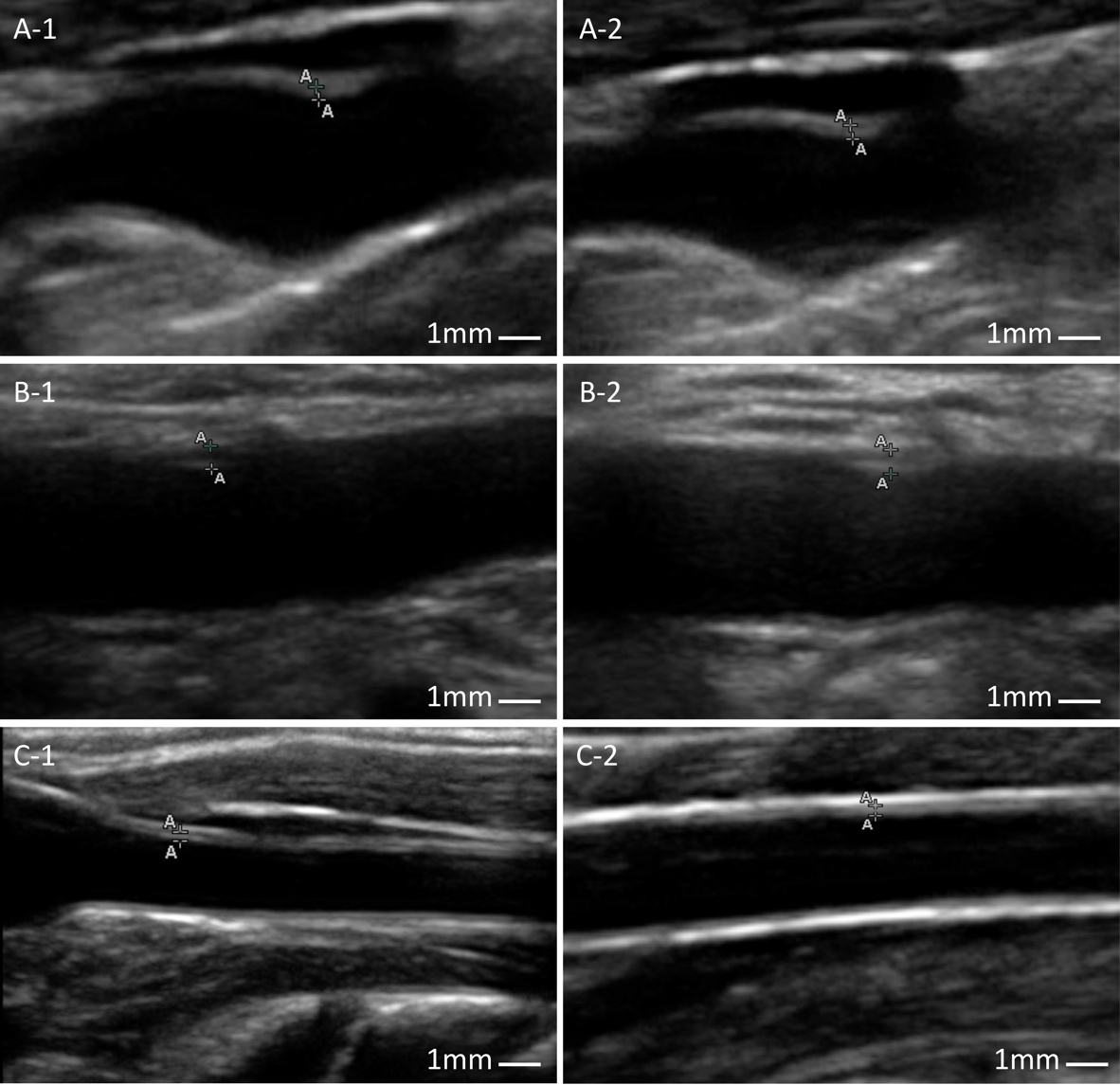
**

**before SCM-198 treatment after SCM-198 treatment**

**Figure S2. Comparison of the changes of artery intima-media thickness (IMT) in different Rhesus monkeys** **before and after the SCM-198 treatment.** A, B and C show images of IMT measurement of right common carotid artery bifurcation (RBIF), abdominal aorta (AO) and left common carotid artery (LCCA), respectively, in different rhesus monkeys before and after the SCM-198 treatment. There are no significant changes in IMT pre- and post- SCM-198 treatment.

**Table S1. Primers used for the study of genes responsible for liver lipid synthesis process including fatty acid synthase (FASN), stearoyl-Coenzyme A desaturase 1 (SCD1) and sterol regulatory element-binding protein (SREBP1, expressed by SREBF1 gene).**

| Gene name | Primer name | Primer sequence (5´ to 3´) |
| --- | --- | --- |
| FASN | mouse_FASN_ F | TTGACGGCTCACACACCTAC |
|  | mouse_FASN_R | TTGTGGTAGAAGGACACGGC |
| SCD | mouse_SCD_F | GAGAAGGGCGGAAAACTGGA |
|  | mouse_SCD_R | GAGCACCAGAGTGTATCGCA |
| SREBF1 | mouse _ SREBF1 _F | AGAGCCCTGCACTTCTTGAC |
|  | mouse _ SREBF1 _R | GTTCAACGCTCGCTCTAGGA |
| GAPDH | mouse _GAPDH_F | GTTTCCTCGTCCCGTAGACA |
|  | mouse _GAPDH_R | GATGGGCTTCCCGTTGATGA |

**Table S2. Comparison of the changes of artery morphology in hypercholesterolemic Rhesus monkeys treated by SCM-198 for 178 days**

| Groups | | Right Common Carotid Artery-Intima Media Thickness  (RCCA-IMT)  (mm) | | Right Common Carotid Artery Bifurcation-Intima Media Thickness  (RBIF-IMT)  (mm) | | Left Common Carotid Artery-Intima Media Thickness  (LCCA-IMT)  (mm) | | Left Common Carotid Artery Bifurcation-Intima Media Thickness  (LBIF-IMT)  （mm） | | | Abdominal Aorta-Intima Media Thickness  (AO-IMT)  (mm) | |  |
| --- | --- | --- | --- | --- | --- | --- | --- | --- | --- | --- | --- | --- | --- |
|  |  | Baseline | End | Baseline | End | Baseline | End | Baseline | End | Baseline | | End | |
| Atorvastatin  (1.2mg/kg) |  | 0.3 | 0.3 | 0.5 | 0.4 | 0.3 | 0.3 | 0.4 | 0.4 | 0.5 | | 0.5 | |
|  |  | 0.4 | 0.4 | 1.0 | 0.9 | 0.4 | 0.3 | 0.5 | 0.4 | Upper segment0.7  Lower segment1.0 | | Upper segment 0.7  Lower segment 0.9 | |
|  |  | 0.3 | 0.3 | 0.4 | 0.4 | 0.3 | 0.4 | 0.5 | 0.5 | 0.5 | | 0.4 | |
| SCM-198 (10mg/kg) |  | 0.4 | 0.3 | - | - | 0.4 | 0.3 | - | - | 0.7  1.2 | | Anterior wall1.2 | |
|  |  | 0.3 | 0.3 | 0.3 | 0.4 | 0.4 | 0.3 | 0.4 | 0.4 | 0.5 | | 0.5 | |
|  |  | 0.4 | 0.3 | 0.5 | 0.5 | 0.4 | 0.3 | 0.5 | 0.5 | Anterior wall 0.6  Bifurcation0.3 | | 0.5 | |
| Control |  | 0.3 | 0.3 | 0.9 | 0.8 | 0.4 | 0.3 | 0.4 | 0.4 | 0.4 | | 0.4 | |
|  |  | 0.4 | 0.4 | 0.5 | 0.6 | 0.3 | 0.3 | 0.5 | 0.4 | Common iliac artery bifurcation0.8  Calcification | | Common iliac artery bifurcation 0.9  Calcification | |

**Table S3. The impact of SCM-198 on the biochemical index of hypercholesterolemic Rhesus monkeys (Mean±SD)**

| Groups | Atorvastatin (1.2mg/kg)  n=3 | | | SCM-198 (10mg/kg)  n=3 | | | Control  n=3 | | |
| --- | --- | --- | --- | --- | --- | --- | --- | --- | --- |
|  | Baseline | End | | Baseline | End | | Baseline | End | |
| ALT/AST | 0.85±0.23 | | 0.72±0.18 | 0.67±0.35 | | 0.92±0.24 | 0.40±0.16 | | 1.07±0.19 |
| GGT (IU/L) | 82.57±15.07 | | 65.45±13.36 | 87.47±25.29 | | 78.77±26.11 | 58.00±11.31 | | 56.60±12.30 |
| ALP (IU/L) | 132.33±37.66 | | 62.05±13.51 | 109.27±37.28 | | 58.47±9.45 | 71.40±10.04 | | 38.80±6.08 |
| Total bilirubin (μmol/L) | 6.37±2.34 | | 6.55±0.78 | 7.10±0.20 | | 5.57±1.25 | 6.45±2.90 | | 8.20±3.96 |
| Direct bilirubin (μmol/L) | 0.73±0.29 | | 0.45±0.21 | 0.83±0.38 | | 0.23±0.23 | 0.70±0.14 | | 1.35±1.20 |
| Indirect bilirubin (μmol/L) | 5.63±2.06 | | 6.10±0.57 | 6.27±0.45 | | 5.33±1.06 | 5.75±3.04 | | 6.85±2.76 |
| Total protein (g/L) | 74.93±6.16 | | 82.00±10.89 | 76.10±2.75 | | 78.97±4.52 | 77.45±2.90 | | 81.65±0.07 |
| Albumin (g/L) | 34.30±4.49 | | 36.30±6.08 | 34.83±1.53 | | 33.07±0.72 | 35.40±4.81 | | 35.45±1.06 |
| Globulin (g/L) | 40.63±3.15 | | 45.70±4.81 | 41.27±2.59 | | 45.90±3.93 | 42.05±1.91 | | 46.20±0.99 |
| Albumin/Globulin (A/G) | 0.85±0.12 | | 0.80±0.05 | 0.85±0.07 | | 0.72±0.05 | 0.85±0.15 | | 0.77±0.04 |
| Urea Nitrogen (μmol/L) | 5.63±0.49 | | 7.40±3.68 | 4.70±0.78 | | 4.40±2.03 | 6.90±1.27 | | 4.95±1.06 |
| Creatinine (μmol/L) | 135.17±68.43 | | 108.25±39.24 | 105.13±22.21 | | 78.67±1.16 | 119.95±56.36 | | 107.10±18.81 |
| Uric acid (μmol/L) | 12.43±1.54 | | 11.20±0.42 | 10.57±0.38 | | 10.73±1.07 | 12.15±0.21 | | 13.85±10.68 |
| Urea Nitrogen/Creatinine | 0.05±0.02 | | 0.08±0.06 | 0.05±0.02 | | 0.06±0.03 | 0.06±0.01 | | 0.05±0.01 |
| CK (IU/L) | 141.87±171.53 | | 199.80±89.10 | 47.93±19.20 | | 64.60±40.16 | 43.00±8.49 | | 60.55±11.81 |

**Table S4. The impact of SCM-198 on the hematological index of hypercholesterolemic Rhesus monkeys (Mean±SD)**

| Group  Parameters | Atorvastatin (1.2mg/kg)  n=3 | | SCM-198 (10mg/kg)  n=3 | | Control  n=2 | |
| --- | --- | --- | --- | --- | --- | --- |
|  | Baseline | End | Baseline | End | Baseline | End |
| RBC cell count (10^12^/L) | 6.55±0.36 | 5.45±0.04 | 3.24±2.77 | 6.23±0.92 | 6.34±0.80 | 5.58±0.33 |
| WBC cell count (10^9^/L) | 5.17±1.33 | 5.80±2.12 | 5.60±4.92 | 6.40±0.95 | 7.80±0.57 | 9.10±2.12 |
| Hemoglobin (g/L) | 138.67±13.50 | 132.00±5.66 | 69.33±60.12 | 149.67±22.68 | 142.50±17.68 | 157.00±9.90 |
| Hematocrit (fL) | 47.50±3.25 | 37.50±3.54 | 23.53±20.39 | 45.13±6.34 | 47.80±6.79 | 41.60±2.69 |
| Mean Corpuscular Volume(fL) | 72.63±5.22 | 68.85±7.00 | 48.63±42.12 | 72.47±0.61 | 75.35±1.20 | 74.55±0.35 |
| Mean Corpuscular Hemaglobin(pg) | 21.20±1.92 | 24.30±1.27 | 14.33±12.41 | 24.03±0.32 | 22.55±0.07 | 28.15±0.21 |
| Mean Corpuscular Hemaglobin Concentration (g/L) | 292.33±8.50 | 353.50±17.68 | 196.33±170.07 | 332.00±5.00 | 299.00±5.66 | 377.50±0.71 |
| Platelet count (10^9^/L) | 177.67±48.42 | 294.00±42.43 | 206.67±187.45 | 238.33±69.41 | 184.00±11.31 | 278.00±25.46 |
| Neutrophil count (10^9^/L) | 2.33±0.91 | 2.30±1.13 | 2.00±1.87 | 3.00±0.36 | 3.10±1.41 | 3.50±2.26 |
| Neutrophil percentage (%) | 44.80±7.02 | 39.40±4.67 | 24.60±24.35 | 47.27±2.16 | 40.85±21.28 | 37.35±16.19 |
| Lymphocyte count (10^9^/L) | 2.10±0.66 | 2.65±0.78 | 2.83±2.67 | 2.47±0.23 | 3.80±2.40 | 4.80±0.42 |
| Lymphocyte percentage (% ) | 41.63±9.42 | 46.95±5.02 | 33.47±29.94 | 38.73±2.42 | 48.35±26.66 | 55.15±17.89 |
| Granulocyte count (10^9^/L) | 0.73±0.25 | 0.85±0.21 | 0.77±0.80 | 0.93±0.38 | 0.90±0.42 | 0.80±0.28 |
| Granulocyte percentage (%) | 13.57±4.57 | 13.65±0.35 | 8.60±8.60 | 14.00±4.25 | 10.80±5.37 | 7.50±1.70 |

Note: atorvastatin (1.2mg/kg), from Day150 (n=2)
